# The Role of Dissolution Time on the Properties of All-Cellulose Composites Obtained from Oil Palm Empty Fruit Bunch

**DOI:** 10.3390/polym15030691

**Published:** 2023-01-30

**Authors:** Mohd Zaim Jaafar, Farah Fazlina Mohd Ridzuan, Mohamad Haafiz Mohamad Kassim, Falah Abu

**Affiliations:** 1 Bioresource Technology Division, School of Industrial Technology, Universiti Sains Malaysia, Penang 11800, Penang, Malaysia; 2 Green Biopolymer, Coatings & Packaging Cluster, School of Industrial Technology, Universiti Sains Malaysia, Penang 11800, Penang, Malaysia; 3 Department of Ecotechnology, School of Industrial Technology, Faculty of Applied Sciences, Universiti Teknologi MARA (UiTM) Shah Alam, Shah Alam 40450, Selangor, Malaysia; 4 Smart Manufacturing Research Institute (SMRI), Universiti Teknologi MARA (UiTM) Shah Alam, Shah Alam 40450, Selangor, Malaysia

**Keywords:** all-cellulose composite, surface selective dissolution, interfaceless, self-reinforced composite, green composite

## Abstract

All-cellulose composite (ACC) films from oil palm empty fruit bunches (OPEFBs) were successfully fabricated through the surface selective dissolution of cellulose fibers in 8 wt% LiCl/DMAc via the solution casting method. The effect of dissolution time on the properties of the ACC films was assessed in the range of 5–45 min. The results showed that under the best conditions, there were sufficiently dissolved fiber surfaces that improved the interfacial adhesion while maintaining a sizable fraction of the fiber cores, acting as reinforcements for the material. The ACC films have the highest tensile strength and modulus of elasticity of up to 35.78 MPa and 2.63 GPa after 15 min of dissolution. Meanwhile, an X-ray diffraction analysis proved that cellulose I and II coexisted, which suggests that the crystallite size and degree of crystallinity of the ACC films had significantly declined. This is due to a change in the cellulose structure, which results in fewer voids and enhanced stress distribution in the matrix. Scanning electron microscopy revealed that the interfacial adhesion improved between the reinforcing fibers and matrices as the failure behavior of the film composite changed from fiber pullout to fiber breakage and matrix cracking. On the other hand, the thermal stability of the ACC film showed a declining trend as the dissolution time increased. Therefore, the best dissolution time to formulate the ACC film was 15 min, and the obtained ACC film is a promising material to replace synthetic polymers as a green composite.

## 1. Introduction

Global attention has been drawn to the urgent appeal for new eco-friendly composite materials to replace petroleum-based ones. According to the 2020 JEC Group report, a non-profit association dedicated to the promotion of composite materials, the global production of composites and plastics currently amounts to 10 and 350 million tons a year, respectively [1]. A challenge now arises in finding technically and economically viable recovery routes for these materials, which were developed on a large scale in the last century and have now become unavoidable. As asserted by the Organization for Economic Cooperation and Development (OECD) in its 2022 report, global plastic waste generation has more than doubled from 2000 to 2019 to 353 million tons [2]. These statistics demonstrate the urgent need to produce novel biocomposite materials with biodegradable properties, such as cellulose, to substitute materials derived from petrochemicals and fossil fuels.

Cellulose is one of the most abundant materials on earth and has eco-friendly features that make it useful as a reinforcement agent for biocomposite materials [3]. However, in most instances, the composite matrix is still frequently based on polymers generated from petroleum [4]. Research into using this bio-based material to create biocomposites has faced obstacles since hydrophilic fibers and hydrophobic matrices cannot coexist due to interfacial adhesion [5,6,7,8,9]. As a result, numerous methods have been used with varying degrees of success to overcome it. These methods include physical and chemical modifications of the fiber and matrix to improve interfacial bonding [10], including silane and alkali treatments, acetylation, chemical grafting, corona discharge, as well as using sub-micron and nano-sized forms of cellulose [11,12]. Although these methods can enhance the mechanical qualities of green composites, they also increase the expense and complexity of their manufacturing [13].

The newly developed green composite called all-cellulose composite (ACC) looks promising for formulating green composites that aim to eliminate the chemical incompatibilities between the reinforcement agent and matrix phases by utilizing cellulose for both components [14,15]. Inspired by the classic mono-component polymer composites developed by Capiati and Porter (1975) [16], Nishino et al. (2004) [17] posed the concept of all-cellulose composites. ACCs can be considered bio-derived mono-component composites, which is the same source of cellulosic materials that would need to be used for the reinforcing and matrix phases. In other words, it is a new class of materials that, unlike other biocomposites, are made entirely from cellulosic materials, leading to chemically identical matrix and reinforcing phases [13]. The following two processing methods are suggested for the manufacture of an ACC: (i) complete cellulose dissolution, followed by mixing with extra reinforcing cellulosic materials, and (ii) partial cellulose dissolution to generate a matrix phase in situ around the residual fiber core [17,18,19,20,21]. Additionally, cellulose-dissolving utilizing solvent is a distinguishing feature of ACC engineering in contrast to other traditional composites due to cellulose’s inherent property, i.e., it does not melt. Lithium chloride/N,N-dimethylacetamide (LiCl/DMAc), NaOH, and ionic liquids (ILs) are some of the most popular solvents used by researchers [13,14]. Despite being environmentally acceptable solvents, it is a challenge to find industrial applications for ionic liquids (ILs) due to their complex and expensive manufacture. On the other hand, toxicity concerns restrict the usage of LiCl/DMAc in academic studies [13].

Although Nishino introduced the first ACC in 2004 by utilizing Ramie fiber, it was only recently that it was given serious consideration. ACCs are mono-material composites made from cellulose. Therefore, they show excellent interfacial compatibility, are fully recyclable, and are environmentally friendly [15,22,23]. As a result, ACCs may represent the next stage in the creation of composite materials with greater sustainability. The world’s primary source of cellulose, forests, would be in peril if cellulose became the only component of this type of composite. For forests to be sustainable, the reliance on plant cellulose, particularly wood, must be significantly reduced through the use of biomass or agricultural waste.

Numerous research has focused on the development of ACCs using non-wood or biomass, including food crops, cotton [23,24], canola straw [25], flax [4], bagasse [26], hemp [14], pineapple leaf [27], Napier grass [28], and many others [24,28,29]. The substantial amount of biomass produced by the palm oil sector is one of the components of this category that has tremendous relevance to the Malaysian situation. In Malaysia, palm oil is a well-known and important commodity [30]. According to the Malaysian Palm Oil Board (MPOB), 400 hectares were cultivated in 1920, leading to five million plantations today in Malaysia [31]. Enormous amounts of lignocellulose residues from the oil palm industry, such as oil palm trunks (OPTs), oil palm fronds (OPFs), and oil palm empty fruit bunches (OPEFBs), are generated at an estimated 15 million tons annually, becoming a major economic pillar for the country [32]. This biomass is readily available at a minimal cost. Attempts have been made to turn these wastes into value-added products; one example is converting the lignocellulosic residue into a paper-making pulp. Hence, other utilization alternatives are sought in an attempt to optimize the use of this biomass. Therefore, the development of ACCs from this biomass would be the best approach.

During the past decade, creating ACC films from OPEFBs has been the subject of only a small number of studies [33,34,35,36,37,38,39]. Several earlier studies had utilized MCCs and nanoscale. However, to date, OPEFBs in the form of bleached pulps, utilizing facile preparation, which is a straightforward pulping and bleaching method, have not been addressed yet. In addition, this form of pure cellulose and simple method can reduce energy and costs and avoid the complexity of ACC preparations. Therefore, the objective of this research is to determine the best dissolution time in an attempt to enhance the performance of ACC films made from OPEFB-bleached pulps. This article reports the effect of dissolution time in the range of 5–45 min on the tensile properties of ACC films. It is expected that the produced ACC films would have the potential to be used for green composite productions and applications.

## 2. Materials and Methods

### 2.1. Materials

Oil palm empty fruit bunches (OPEFBs) were obtained from United Oil Palm Sdn Bhd, Nibong Tebal, Penang, Malaysia. All of the reagent-grade chemicals, including N-N dimethyl acetamide (DMAc), lithium chloride (LiCl), sodium hydroxide (NaOH), methanol, and acetone, were derived from Nacalai Tesque Co., Kyoto, Japan, and used as received.

### 2.2. Isolation and Extraction of OPEFB Cellulose

In this work, non-cellulosic components were removed from cellulose samples made from OPEFB biomass sources. By using soda pulping, extended delignification by bleaching, and hemicellulose removal, cellulose was obtained from the OPEFBs. Based on the processes disclosed by Wanrosli et al. [40], the pulping and bleaching sequence of the OPEFB pulp was carried out. To isolate the cellulose, the dried fiber was first delignified according to ASTM D 1104-56 to produce holocellulose, followed by the removal of the hemicellulose fraction according to ASTM D 1103-60.

#### 2.2.1. Soda Pulping Process

The pulping process was achieved through the use of a digestor (model IBSUTEK ZAT 92) from RB Supply Enterprise, Penang, Malaysia. The OPEFB was cooked at 26% NaOH with a ratio of 1:8 (OPEFB: liquor) at 170 °C for 2 h. A hydropulper was then used to refine the pulp, which was then washed with water, screened, dried, and noted as OPEFB-P.

#### 2.2.2. Holocellulose Production

A total of 5 g of pulp from the OPEFB-P was transferred into a 1000-mL conical flask, and 160 mL of distilled water, 1.5 g of sodium chlorite (NaClO_2_), and 10 drops of acetic acid (10% wt/v) were added. The sample was kept inside a water bath at 70 °C for 3 h. Next, 1.5 g of NaClO_2_, 10 drops of acetic acid, and 160 mL of distilled water were added alternately for each hour. After 3 h, cooled distilled water was added to stop the chemical reaction in the flask. The precipitate was then filtered with a glass crucible (2G2). The remaining pulp in the glass crucible was washed using cold distilled water, followed by acetone, and it was left to dry in the oven for 24 h.

#### 2.2.3. Bleaching Process

The 2.0 g of the holocellulose (hemicellulose + cellulose) produced was treated with 50 mL of 17.5% sodium hydroxide at 20 °C for 2 h, and then 70 mL of distilled water was added. This was to separate the hemicellulose from the holocellulose, leaving the cellulose. The insoluble cellulose was filtered and washed with 50 mL of 8.3% sodium hydroxide.

### 2.3. Preparation of All-Cellulose Composite Film

In another article, the surface selective dissolution procedure used to create all-cellulose composites was described [19,41,42]. The OPEFB pulp was immersed in distilled water, acetone, and DMAc, respectively, for 1 h each at room temperature to activate the cellulose fibers. The activated EFB pulp was immersed in the LiCl/DMAc solution at room temperature for four different dissolution times (5, 15, 30, and 45 min) with a 1% initial cellulose concentration (wt. vol-1). The solution was then cast on a glass plate with a blade and allowed to dry for 24 h in the open air. After that, the obtained ACC was immersed in distilled water for 24 h and further dried in the open air overnight.

### 2.4. X-ray Diffraction

The Bruker D2 Phaser X-ray diffractometer (XRD), Bruker, Germany, equipped with the LYNXEYE 1D solid-state ultra-fast detector with Cu-Kα radiation at room temperature was used to analyze the crystallinity of the ACC films. The samples were analyzed using a step size of 0.02 and a dwell time of 0.1 s per step. The following equation was used to calculate the crystallinity index (CrI) for all ACC composite films [35]:

Crystallinity Index (CrI) = ( I − I’ / I ) / ( I ) × 100

where I = the height of the peak assigned to (200) planes, measured in the range 2θ = 20–23°, and I’ = the height of the peak assigned to (100) planes, located at 2θ = 12–16°.

### 2.5. Fourier Transform Infrared (FTIR) Analysis

Fourier transform infrared (FTIR) spectroscopy was performed using a Nicolet Avatar Model 360 spectrometer (Thermo Nicolet Corporation, Madison, WI, USA). The FTIR analysis was measured in the transmittance mode from 500 nm to 4000 nm.

### 2.6. Scanning Electron Microscopy (SEM)

The tensile fracture surfaces of the composite samples were examined by scanning electron microscopy (Quanta FEG 650, FEI, Brno, Czech Republic). The samples were placed on the SEM holder using double-sided electrically conducting carbon adhesive tape and gold-coated with a Polaron SEM coating unit. The SEM micrographs were obtained under conventional secondary imaging conditions with an acceleration voltage of 10 kV.

### 2.7. Tensile Testing

Tensile tests were conducted according to ASTM D 882 using a texture analyzer (TA.XT plus, Stable Micro Systems, Surrey, UK) with sample dimensions of 150 mm × 600 mm (width × length). The samples were equilibrated in a desiccator at room temperature with an RH range of 30–50% prior to the tensile testing. The tensile strength (TS), Young’s modulus (E), and elongation at break (EAB) were recorded. The filmstrip was clamped between the tensile grips with an initial grip separation of 100 mm, and the test speed was set at 0.8 mm s ^−1^ using a load cell of 30 kg. An average of five replicates were recorded.

### 2.8. Differential Scanning Calorimetry (DSC)

A differential scanning calorimetry analysis was performed using a Perkin Elmer Pyris 7 thermal analyzer (Radeberg, Germany). About 6 mg of the sample were put into the sample pan, after which they were sealed and heated at 10 °C/min in a nitrogen flux from 30 to 400 °C. The data obtained from the samples were recorded continuously along with the temperature and time intervals.

### 2.9. Thermogravimetric Analysis (TGA)

About 10 mg of the sample was heated at 10 °C/min in a nitrogen flux (20 mL/min) from room temperature to 800 °C in a nitrogen flux atmosphere using a Mettler Toledo TGA/DSC 1 (STAR e system) (Schwerzenbach, Switzerland). The percentage of sample weight loss versus temperature was recorded using a thermogram.

## 3. Results and Discussion

Following this point, the samples were identified by their initial cellulose concentrations (preceded by “ACC”) and their dissolution times (preceded by “5–45”). The initial cellulose concentration for each of the other ACCs is set to 1% (wt.vol^−1^) to ensure a single variable. For example, sample ACC5 designates a composite prepared with a dissolution time of 5 min in an 8% LiCl/DMAc solution. Table 1 summarizes the formulation of the all-cellulose composite films in the present work.

### 3.1. Tensile Properties

**Figure 1:**
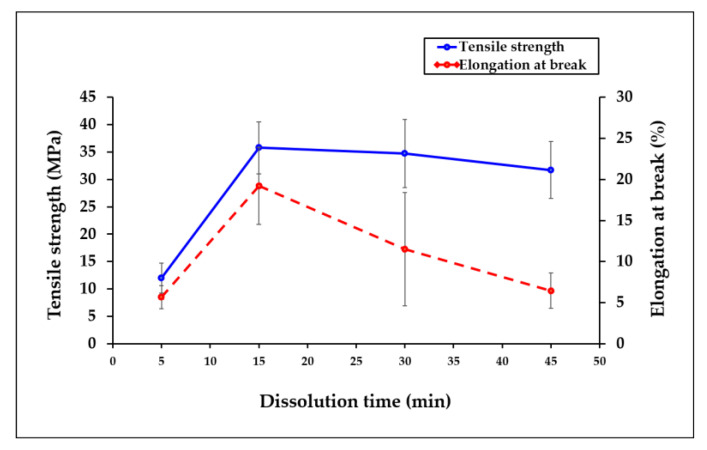
Mechanical properties of the ACC film composites made from OPEFB at dissolution times ranging from 5 to 45 min.

Figure 1 shows the effect of dissolution time on the tensile strength (TS) of all-cellulose composite films. The mean values of the tensile strength, elongation at break (EAB), and modulus of elasticity are shown in Table 2. Overall, it can be observed that the longer the dissolution time, the higher the TS of the ACC films. The results showed that the TS of the initial ACC5 increased from 11.96 MPa to 35.78 MPa at its highest when the dissolution time reached 15 min, increasing by 66.58% in comparison to the initial film with a 5-min dissolution period. This finding is in line with the results reported for ACCs from other cellulose sources, such as flax, cotton fiber, canola straw, pineapple leaf, bacterial cellulose, and bagasse, in response to the impact of longer dissolution times that lead to significant improvements in the mechanical behavior of ACC films [4,24,25,27,42]. The tensile properties of the ACCs generated from those cellulose sources were considerably altered with the longer dissolution times, up to an ideal period.

The properties of the ACC films depend on the matrix, reinforcing phase, and interface [22]. The improvement in the ACC films also filled the voids between the fibers and cracks, which is probably because of the dissolved matrix [4,15,43]. This suggests that the reinforcing phase and the polymer matrix interact strongly, preventing the mobility of polymer chains. Furthermore, because of the improved contact between the fibers, the strain at break of the all-cellulose composites was observed to significantly rise with the longer dissolution periods. As a result, there was an increase in the process of stress transfer from the matrix to the reinforcing material, hence, fostering the TS traits [27].

In contrast, the TS of the films showed a slight drop from 35.78 MPa to 31.72 MPa when the dissolving time was prolonged from 15 to 45 min, although the TS value remained superior to the initial film with the 5-min dissolution time. The excessive dissolution of cellulose reduces the fraction of the reinforcing phase and, hence, weakens the mechanical properties [22]. As the dissolving time increases, the TS of the ACC film decreases due to its high wettability [41,42]. Similar to the findings of Yousefi et al. (2011) [25], the TS decreases when the dissolution time is prolonged due to the aggregation of the cellulose particles. Cellulose cannot perform its fundamental function when the particles are gathered. Thus, the TS typically decreases.

Due to the various features that affect the EAB and TS values, the EAB trend typically opposes the TS trend for many types of composites. EAB measures a material’s ductility, while TS provides information about a material’s strength. The addition of more fillers would typically raise the composite’s rigidity in a conventional composite system, reducing the material’s elongation at break (flexibility). In the ACC system, higher dissolution times result in increased matrix production and a lower reinforcing phase volume. As a result, according to typical composite results, the EAB result should decrease as the dissolution time increases. In this study, however, it is intriguing to note that EAB exhibits a directly proportionate tendency to TS, which is somewhat raised when the dissolution time is prolonged from 5 to 15 min before gradually decreasing as time passes.

This might be explained by the fact that the ACC was created using only one material, EFB cellulose, which served as both the matrix and the reinforcement. This resulted in an uncontrollable amount of dissolved (for the matrix) and undissolved (for the reinforcement) cellulose in the structure of the ACC films, also known as the fiber volume fraction. Estimating the fiber volume fraction of ACCs made using partial dissolving techniques presents various difficulties. In contrast to conventional fiber-reinforced composites, the amount of fiber needed to make ACCs will not be the same after processing because some of the fiber will have dissolved to become the matrix [26].

In this study, the fiber volume fraction is inferred rather than measured by varying the dissolving time; the higher the fiber volume percent produced, the longer the dissolution time. Consequently, the fiber volume fraction produced the matrix and reinforcement ratio that affected the EAB outcome. Despite the dissolving time being prolonged to 45 min, there was not enough reinforcing phase inside the matrix domain to permit appreciable changes in the elongation at break of the ACC films. As a result, the fraction that contributes to elasticity may also change since the “actual composition” of the reinforcing phase in this situation may not be proportional to the EFB content.

In addition to the variable elongation at break value, environmental factors including moisture absorption, film absorption rate, and varying void contents due to the random dispersion of the EFB particles in the ACC film structures could also be factors. These elements have an impact on the biopolymer chains’ flexibility and conformation when they are deformed by tensile forces. These explanations could account for the surprising elongation at break results of these ACC films [39].

**Figure 2:**
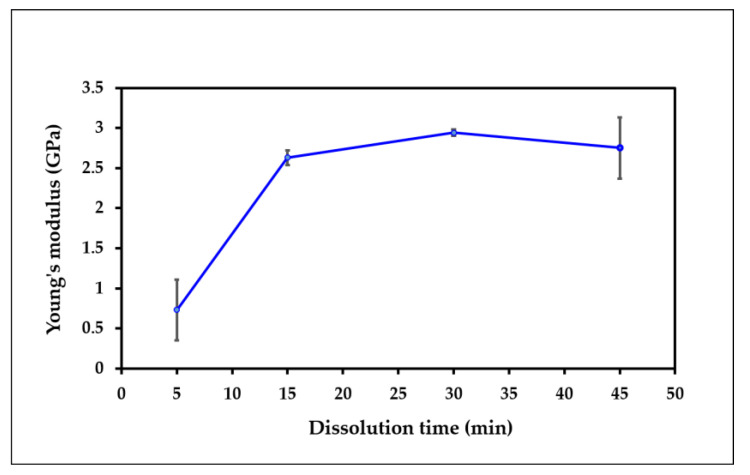
Modulus of elasticity of the ACC films at various dissolution times (5–45 min).

Figure 2 shows the effect of dissolution time on the modulus of elasticity of the all-cellulose composite films. The modulus of elasticity value increases for up to 30 min of the dissolution time and then drops afterwards. The reason for the decrement could probably be due to the increase in the OPEFB contents, which can cause aggregation of the particles. Additionally, the particles that were not dispersed well in the solvent system were reported by Zailuddin et al. [35].

### 3.2. FTIR Analysis

**Figure 3:**
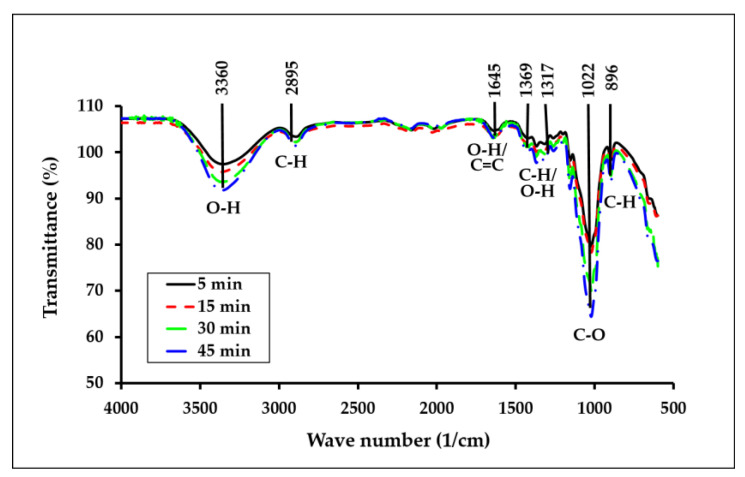
FTIR spectra of the ACCs film composites at different dissolution time.

FTIR spectroscopy was used to distinguish between the cellulose allomorphs and the celluloses of different crystallinities. In Figure 3, the FTIR spectra of the understudied samples plotted in absorbance form within the range of 4000–500 cm^−1^ are shown. The effect of the dissolution time on the structural changes of the ACC films was analyzed using FTIR, which reflects the changes in the functional groups. It can be observed that all four spectra had common bands, as both the matrix and the reinforcement agent in the ACCs are cellulosic. All samples displayed two main absorbance regions and are found at high (3000–3600 cm^−1^) and low (500–1800 cm^−1^) wavenumbers, respectively.

Table 3 shows the list of characteristic FTIR peaks and the corresponding motions of the organic bonds in this study. Based on the spectra, it is evident that the O–H groups, which make up the majority of the OPEFB ACC films, reside in a broad absorption band in the 3700–3100 cm^−1^ area. Besides that, the broad peak in the wavenumber range of 3000–3600 cm^−1^ is believed to be generated by the O(3)H–O(5) intramolecular hydrogen bond [44], and the peak at about 2850 cm^−1^ is attributed to the stretching vibration of C–H bonds in the methyl and methylene groups [45]. The peak located at about 1420 cm^−1^ is assigned to methyl group deformation and the lignin aromatic ring vibrations, whereas the peak at about 1255 cm^−1^ is associated with C–O stretching in the guaiacyl ring [27]. The peaks at 1310 cm^−1^ and 1370 cm^−1^ are respectively assigned to the CH_2_ wagging motion and the –OH in-plane bending of the crystalline form of cellulose [44]. The peaks centered at about 1160 cm^−1^, 1025 cm^−1^, and 895 cm^−1^ are attributed to C–O–C asymmetric bridge stretching, C–O–C pyranose ring skeletal vibration, and β-glucosidic linkage, respectively [27]. These peaks are attributed to cellulose II [27], and the increase in their intensities results in more formation of the structure. It was claimed that the band of the O–H region (3000–3600 cm^−1^) contrasts strongly with that of the materials produced by partial dissolution [44]. Accordingly, the monotonic shift of the band to lower wavenumbers by increasing the dissolution time indicates the generation of more new inter- and intramolecular hydrogen bonds, meaning a higher transformation from cellulose I to cellulose II [46]. It is well known that cellulose II is thermodynamically more stable than cellulose I and exists in antiparallel strains with intersheet hydrogen bonding [47].

**Figure 4:**
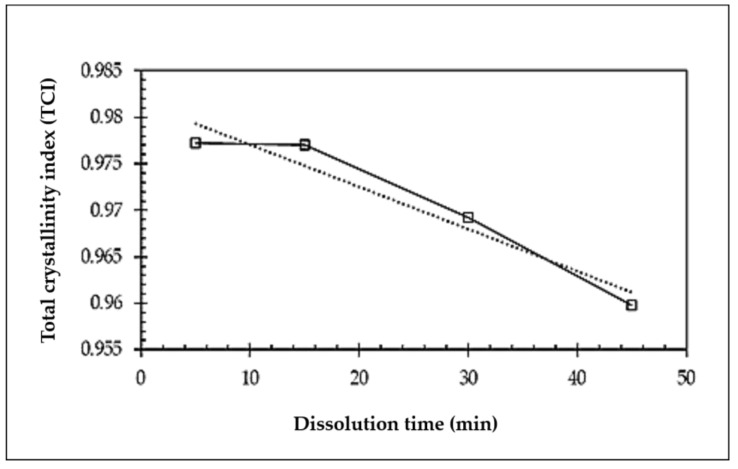
TCI variations obtained from the ratio of the absorption peaks at 1370/2850 cm^−1^ for the understudied samples.

The calculation of the total crystallinity index (TCI) was performed from the ratio of the absorption peaks at 1370/2850 cm^−1^ [48]. These two peaks were chosen because of their low susceptibility to water. The variations of the indices for the understudied samples are shown in Figure 4.

For instance, the understudied samples have TCI values in the range of 0.95–0.98. Nelson and O’Connor [49] reported a value of 0.84 for hydrolyzed cotton cellulose and a value of 0.90 for purified cotton yarn. The same authors reported values for partly mercerized cotton in the 0.54–0.58 range [50]. Carillo et al. [48] reported values in the 0.64–0.87 range for regenerated fibers of different origins, whilst Duchemin et al. [44] reported values in the 0.66–0.95 range for filter paper (FP) and micro-fibrillated cellulose (MFC). From the TCI value variations, it can be seen that the parameter has a decreasing trend versus the dissolution time. The sample underwent a monotonic TCI loss, starting from the highest value of about 0.98 initially and decreasing to a value of about 0.96 after 45 min. It is in good agreement with the TGA data and shows that the sample with a lower initial concentration (1%) is sensitive to the dissolution time. Hence, the FTIR results confirmed that the structural change of cellulose from cellulose I to cellulose II can be detected in the composites by increasing the dissolution time in the samples.

In order to get more information about the structure of the materials and the crystal structure of the ACCs, the ACC films prepared at various dissolution times were examined by X-ray diffraction.

### 3.3. X-ray Diffraction (XRD)

**Figure 5:**
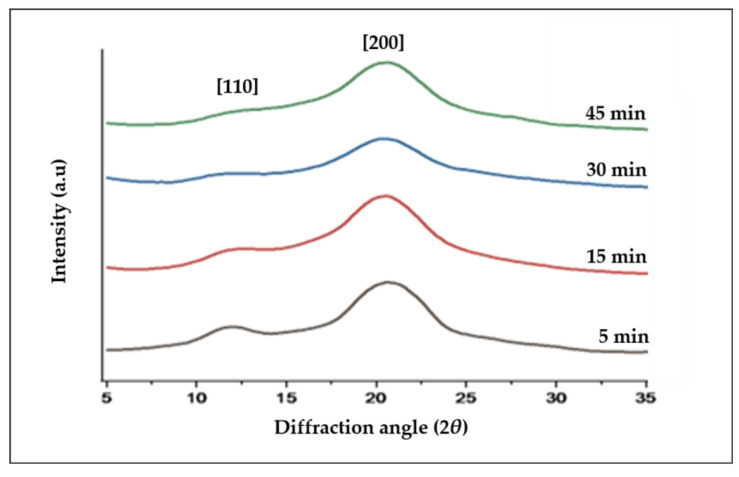
XRD patterns of the ACC film composites at different dissolution time.

The XRD curve of the all-cellulose composite films with different cellulose contents and at dissolution times is presented in Figure 5. The X-ray diffraction (XRD) analysis suggests that the degree of crystallinity of the ACC films significantly declined as the structure of the dissolved cellulose changed from cellulose I to cellulose II.

All ACC samples showed the characteristic peaks at 2θ ≈ 12.0° for the (1 0 1) plane, 2θ ≈ 13° for the (1 0 1) plane, and 2θ= 23.0° for the (0 0 2) plane [14]. For qualitative analysis of the XRD patterns, crystallinity index (CrI) evaluations were calculated by the following equation (Equation (1)) [51]:

Crystallinity Index (CrI) = (I − I’) / ( I ) × 100(1)

where I is the diffraction intensity assigned to (2 0 0) plane of cellulose I_β_ (2θ ≈ 23°). I′ is the intensity measured at 2θ = 18°, where the maximum happens in a diffractogram for the non-crystalline cellulose. Crystallite size (width) of the cellulose was assessed by Scherrer’s equation (Equation (2)) [52]:

D = Kλ / βcos (θ) (2)

where D is the crystal size, K is Scherrer’s constant (0.9), λ is the X-ray wavelength (0.15418 nm), θ is the Bragg angle for the (2 0 0) reflection and β is the corrected integral width.

**Figure 6:**
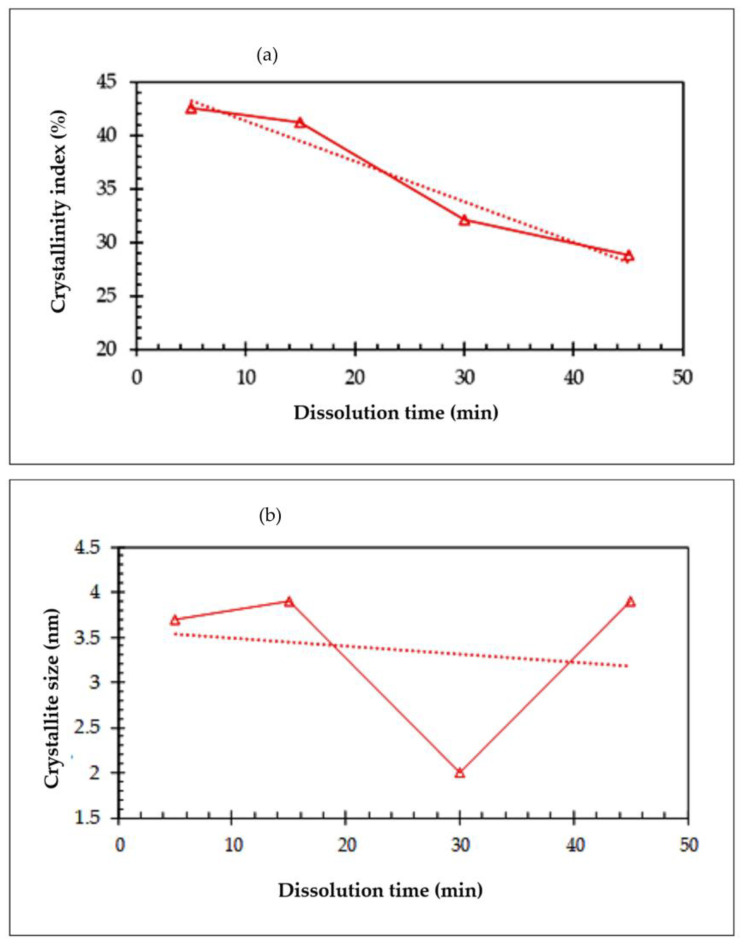
The variations in crystallinity index and crystallite size. (**a**) Crystallinity index; (**b**) Crystallite size.

A comparison of the X-ray diffraction patterns of the all-cellulose composites prepared with 5, 15, 30, and 45 min of dissolution time shows that the crystalline peak intensities of the composites mostly decreased with the dissolution time. The intensity decrement trend with increasing dissolution time is clearer for the crystalline peaks of ($1\;\bar{1}\;0$) and (1 1 0) plans in the 2θ range of 10–15°. In fact, at longer dissolution times, larger fractions of the fibers are dissolved to form an amorphous matrix phase (non-crystalline domains). This confirms the findings of previous studies [6,24,53,54] and indicates that the increase in dissolution time would result in a transformation of cellulose I to cellulose II, as demonstrated by the FTIR analysis. This phenomenon directly results in a reduction in the overall crystallinity index and crystallite size of the composites, which were calculated from the XRD data, as shown in Figure 6 and tabulated in Table 4.

According to Figure 6a, the highest crystalline index values in almost all dissolution times belong to the sample with the initial dissolution time, which was 5 min. In addition, from this figure, it can be seen that the rate of the CrI loss for the sample was significant, indicating a higher sensitivity to the dissolution time, as seen in the TGA and FTIR results. The decreasing trend of the CrI parameter with increasing the dissolution time can be attributed to the effect of more solvents penetrating the spaces between the crystallites and dissolved fibers, as well as the outer chains of the crystallites [25]. 

The processing of the X-ray diffraction data using the Scherrer’s equation (Figure 6b) also reveals that the lateral crystallite size normal to the (2 0 0) plane in cellulose composites is reduced with the immersion time. In other words, for all the ACC samples prepared with dissolution times of 5, 15, 30, and 45 min, more crystalline cellulose is dissolved and, hence, smaller cellulose crystallites remain with larger non-crystalline domains being formed. For the ACC30 crystallite size, which is the smallest size, there is an interesting consequence. It is possible that the fiber inside sample ACC30 has a smaller diameter or fewer entanglements than those in the other samples. As a result, the unusually deep penetration of the solvent caused it to become amorphous more quickly and lose more crystallite size. This was also illustrated by the FTIR spectra of the composites (Figure 3), which indicate that the absorption peak intensity of cellulose II increased with the dissolution time.

### 3.4. Scanning Electron Microscopy (SEM)

**Figure 7:**
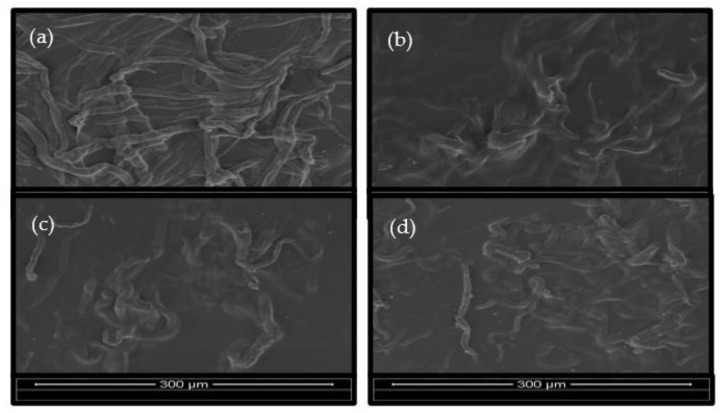
SEM micrographs of the surface morphology of the all-cellulose composites at different dissolution times: (**a**) ACC5, (**b**) ACC15, (**c**) ACC30, and (**d**) ACC45. (magnification ×500).

Figure 7a–d presents the SEM images of the surface of the ACC films as a function of the dissolution time. The matrix was created from the fibers’ outer portions and joined nearby fibers together after a 5-min dissolving period, as it can be clearly seen. Due to insufficient wetting of the fibers at this early stage, the TS value is the lowest compared to others, as predicted, and it is still possible to see microfibers that have not yet dissolved.

Theoretically, with the increase in the dissolution time, larger amounts of the matrix were formed, as reported [23,55]. At 15 min of immersion, there was a skyrocketing increase in the TS value. This can be explained by the formation of enough matrix from the disintegration of cellulose to cover the spaces between the fibers. In the SEM images, there is a noticeable reduction in the outlines and sinking of the fibers’ “vein”. However, the TS value gradually decreased as the dissolution time increased. This is due to the films becoming more brittle as a result of the excessive amount of matrix created. Hence, the key to understanding the failure behavior lies in the optimum 15-min dissolution time. This SEM analysis supports the TS results, whereby the dissolution time in the range of 15–45 min has a higher tensile strength compared to the 5-min initial dissolution time.

Morphology studies using SEM confirmed the XRD and TS results, i.e., that the coexistence of cellulose I and II enhanced the interfacial adhesion between the reinforcing fibers and matrices as the failure behavior of the film composite changed from fiber pullout to fiber breakage. In addition, a better surface consists of fewer voids.

**Figure 8:**
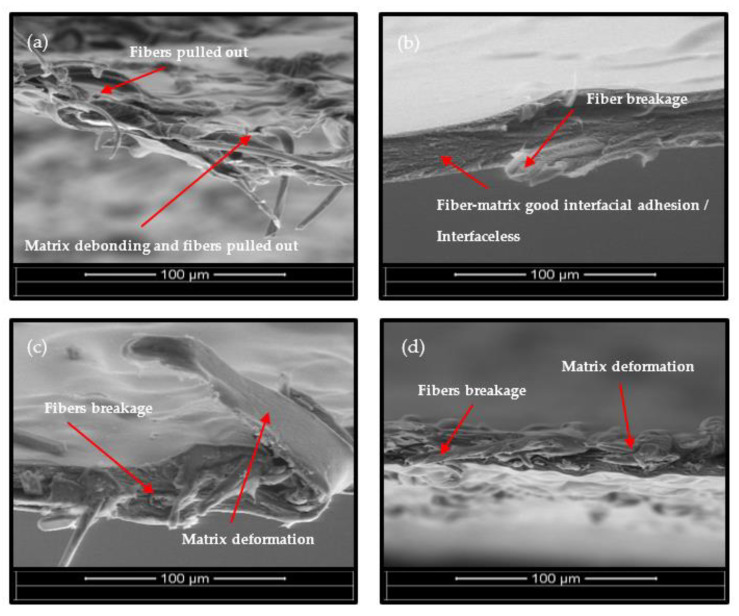
Cross-sectional SEM images of all-cellulose composite films at different dissolution times: (**a**) ACC5, (**b**) ACC15, (**c**) ACC30, and (**d**) ACC45. (magnification ×500).

The study of the cross-sectional characteristics of ACC composite films can provide a fair indication of the degree of interfacial adhesion between the fibers in a composite. In this case, the composites prepared with longer dissolution times exhibit better TS values. The fracture surfaces of the ACC films after tensile deformation are shown in Figure 8. Following the five minutes of dissolution, the microvoids and fiber pullout deformation are visible, as shown in Figure 8a. At the dissolution period of five minutes, the matrix (dissolved cellulose) was unable to fill all the gaps between the fibers and all adjacent fibers. Thus, the mechanical characteristics of the composites started to lower due to the inefficient stress transfer from the matrix to the reinforcement. This failure mechanism has been reported for cellulose mats with high porosity and weak interfiber bonding [27]. The ACC films showed a smoother and more homogenous surface at the dissolution time of 15 min, which is the greatest TS value of any other film. Therefore, stress can be efficiently transferred from the matrix to the reinforcement (undissolved cellulose microfibers). However, the failure mode changed to fiber breakage after the 15-min dissolution time, showing higher mechanical properties resulting from the better interaction between the microfibers and the cellulose matrix.

### 3.5. Thermogravimetric TGA

**Figure 9:**
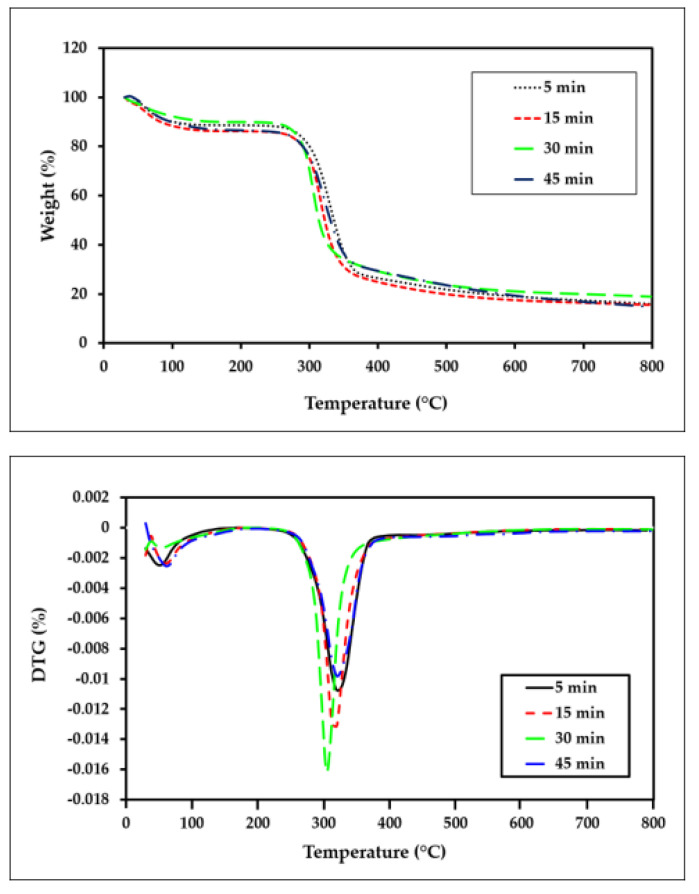
TGA−DTG curves of all-cellulose composites after 5, 15, 30, and 45 min of dissolution.

In order to examine the influence of the dissolution times on the thermal stability of the film composites, a thermogravimetric analysis of the OPEFB cellulose at different dissolution times in the temperature range of 30–800 °C was performed. Figure 9 presents the TGA/DTG curves of the all-cellulose composites at different dissolution times.

**Figure 10:**
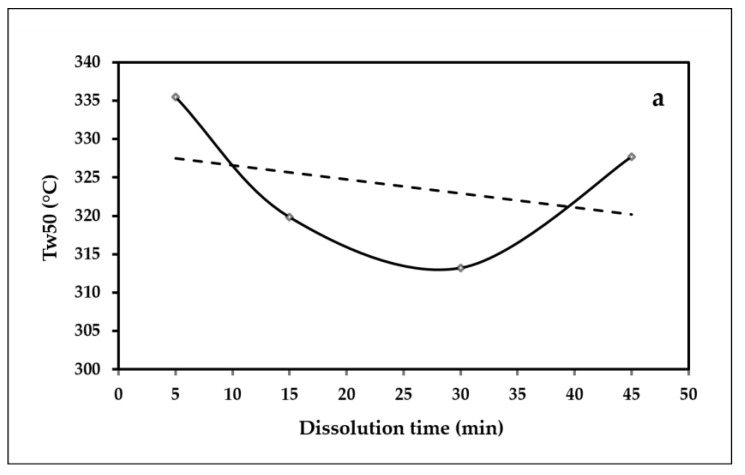

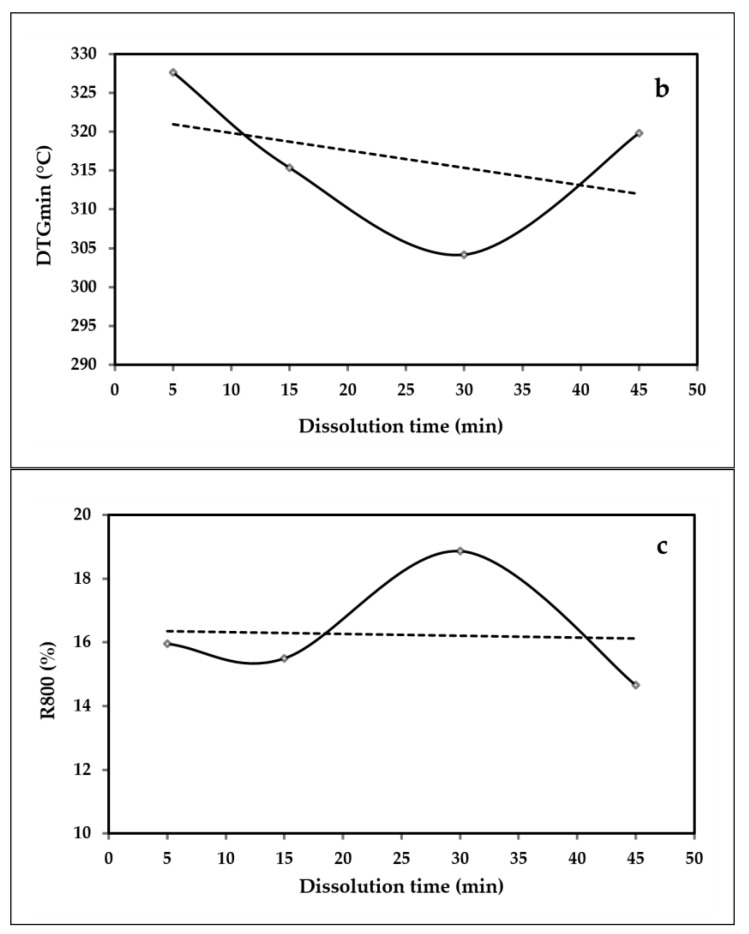
The variations in (**a**) the 50% weight loss temperatures (T_w50_), (**b**) the temperature of the minimum point of the DTG peak (DTG_min_), and (**c**) the amount of residual char at 800 °C (R_800_).

The weight loss in the early temperature stage (<100 °C) can be attributed to the evaporation of moisture physically adsorbed to the ACC structure [27,56]. The weight loss for all types of ACC films remained nearly unchanged at temperatures between 100 and 240 °C. This could be due to the removal of the remaining hemicellulose and extractives during the pulping and bleaching processes and the fiber isolation and extraction. Meanwhile, the sharp drop in weight in the 250–350 °C temperature range corresponds to the loss of crystal water and the thermal degradation of the main cellulose materials [24]. The variations in the amount of residual char of the ACC samples at 800 °C (R_800_), the 50% weight loss temperatures (T_w50_) of the samples, and the temperature of the minimum point of the DTG peak (DTG_min_) are shown in Figure 10; the value is also included in Table 5.

It is clear from Figure 10a,b that the general trends of the T_w50_ and DTG_min_ are decreasing, and longer dissolution times lead to a decrease in the temperatures, as previously indicated by other researchers [24,26,27]. This is because the long dissolution time results in an almost complete dissolution of the cellulose fibers. On the other hand, the general variation trends, shown in Figure 10c, indicate that the amount of residual char at 800 °C increased with increasing the dissolution time. The increased char residue due to the increased dissolution time can be related to the fact that the surface of the cellulose structure exhibits flame retardant properties, which acted as a catalyst for the dehydration and thermal degradation of the ACCs, resulting in the increased char residue in the presence of more cellulose-based structures (lower dissolution times) [57].

### 3.6. Differential Scanning Calorimetry (DSC)

**Figure 11:**
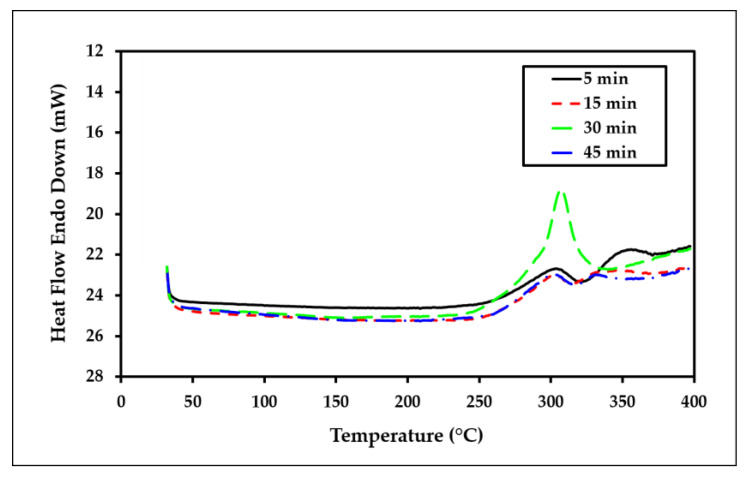
DSC thermograms of the all-cellulose composites after 5, 15, 30, and 45 min of dissolution time.

Figure 11 shows the DSC thermograms of the understudied samples from 30 °C to 400 °C. The endothermic peak in the temperature range of 250–370 °C was attributed to cellulose melting, which corresponded to the breakage of glycosidic bonds and the depolymerization of the cellulose [58]. This indicated that the molecular structure had changed, as observed in the TGA curves, and was also related to thermal decomposition. Moreover, the thermal transitions in this temperature range are associated with the onset of thermal degradation of the cellulose, which consists of the rearrangement of molecular chains, followed by molecular chain cleavage of glucosidic bonds and intermolecular cross-linking [59]. This is in agreement with the work of Miranda et al. [60], which used simultaneous differential thermal analysis (simultaneous DSC-TGA).

**Figure 12:**
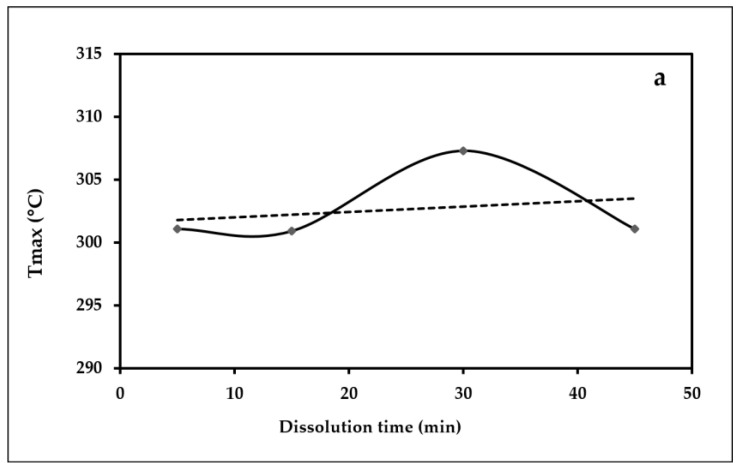

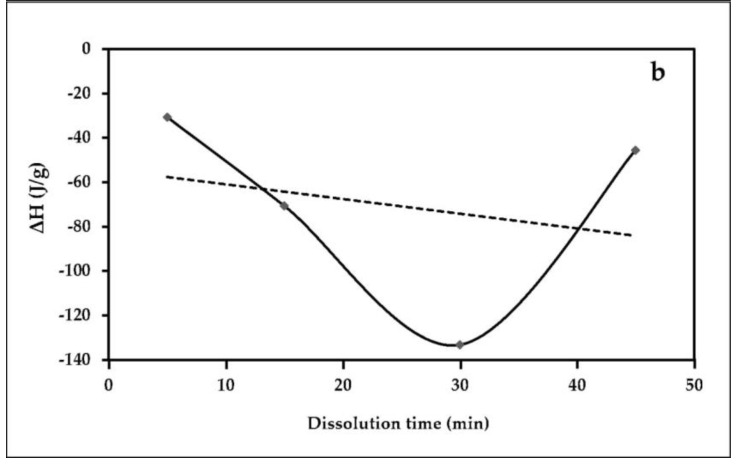
The variations in (**a**) T_max_ and (**b**) ∆H versus dissolution time.

Figure 12 presents the variations of the maximum point temperature of each peak and the obtained ∆H from the DSC diagrams versus the dissolution time for the samples. The values are tabulated in Table 6. According to Figure 12a, the general trend of T_max_ decreases with increasing the dissolution time of the samples. It can be explained by the breakage of more intramolecular and intermolecular hydrogen bonding in the molecular chain during the dissolution process, resulting in a decrease in crystallinity and an endothermic transition. Besides that, Liu et al. [61] also identified that regenerated cellulose films have a lower endothermic peak than the original cotton pulp due to the breakage of hydrogen bonds. From Figure 12b, it is clear that the dissolution time has no significant effect on the ∆H values.

## 4. Conclusions

The relationships between the dissolution time and the properties of the all-cellulose composites obtained by the partial dissolution of OPEFBs in LiCl/DMAc were successfully investigated. The following are the main findings that can be summarized from the performed analyses:

- The amount of dissolved fiber surfaces is adequate to provide sufficient interfacial adhesion to the composite, while a considerable fraction of the fiber cores remain, reinforcing the material.
- The best dissolution time was discovered to be 15 min, which has the highest tensile strength.
- A decrease in the crystallite size and degree of crystallinity was observed with an increase in the dissolution time in the ACC films. The initial crystallinity of the OPEFB-bleached pulp affected the processing and the properties of the all-cellulose composites.
- The thermal stability of the ACC films shows a declining trend as the dissolution time is increased.

The best composite produced in this study has comparable tensile qualities to other ACC films described in past studies, making it a good candidate to be used in the production of green composites.

## Figures and Tables

**Table 1:** Formulation of the all-cellulose composite films in the present work under different conditions.

Samples	Dissolution Time, td (min)	Initial Cellulose Concentration, C (%)
ACC5	5	1
ACC15	15	1
ACC30	30	1
ACC45	45	1

**Table 2:** Mechanical properties of the all-cellulose composite films prepared at different dissolution times.

ACC Films	Tensile Strength (MPa)	Young’s Modulus (GPa)	Elongation at Break (%)
ACC5	11.96 ± 2.7	0.73 ± 2.1	5.66 ± 1.4
ACC15	35.78 ± 4.7	2.63 ± 2.2	19.22 ± 4.7
ACC30	34.72 ± 6.3	2.94 ± 4.1	11.53 ± 6.9
ACC45	31.72 ± 5.2	2.75 ± 1.2	6.46 ± 2.2

**Table 3:** Characteristic FTIR peaks and corresponding motions of organic bonds.

Wave Number (cm^–1^)	Bond and Motion
3600–3000 2850 1420 1370 1310 1255 1160 1025 895	O(3)H–O(5) intramolecular hydrogen bond stretching vibration of C–H bonds in methyl and methylene groups methyl group deformation and the lignin aromatic ring vibrations –OH in-plane bending of crystalline form of cellulose CH_2_ wagging motion C–O stretching in guaiacyl ring C–O–C asymmetric bridge stretching C–O–C pyranose ring skeletal vibration β-glucosidic linkage

**Table 4:** Degree of crystallinity and crystallite size of the ACCs of the ACC films.

Sample	Degree of Crystallinity (%)	Crystallite Size (nm)
ACC5	42.62	3.7
ACC15	41.21	3.9
ACC30	32.08	2
ACC45	28.8	3.9

**Table 5:** Thermal properties of the ACC films.

Sample	Degradation Temperature, °C T_w50_	DTG_min_, °C	Residual Char at 800 °C, % R_800°C_
ACC5	335.5	327.66	15.95
ACC15	319.83	315.34	15.49
ACC30	312	304.17	18.86
ACC45	327.66	319.83	14.66

**Table 6:** The variations in (a) T_max_ and (b) ∆H versus dissolution time.

Sample	Maximum Point Temperature, °C T_max_	Enthalpy, (J/g) ∆H
ACC5	301.09	−30.79
ACC15	300.91	−70.53
ACC30	307.32	−123.24
ACC45	301.07	−45.64
